# Smoking and long-term risks for morbidity and mortality after coronary artery bypass grafting

**DOI:** 10.1016/j.ijcrp.2025.200512

**Published:** 2025-09-08

**Authors:** Emelie Johansson, Malin Stenman, Emma C. Hansson, Carl-Johan Malm, Sossio Perrotta, Aldina Pivodic, Anders Jeppsson, Susanne J. Nielsen

**Affiliations:** a Department of Molecular and Clinical Medicine, Sahlgrenska Academy, Gothenburg University, Gothenburg, Sweden; b Department of Cardiology, Sahlgrenska University Hospital, Gothenburg, Sweden; c Perioperative Medicine and Intensive Care Function, Karolinska University Hospital, Stockholm, Sweden; d Department of Molecular Medicine and Surgery, Karolinska Institute, Stockholm, Sweden; e APNC Sweden, Mölndal, Sweden

## Abstract

**Background:**

We explored the association between smoking and long-term risks of morbidity and mortality in patients who had undergone coronary artery bypass grafting (CABG).

**Methods:**

This population-based registry study included 27,434 patients (mean age 67.9 years, 18.2 % women), divided into: never smokers (n = 8,593), former smokers (n = 14,666) and current smokers (n = 4,175), who underwent CABG between 2010 and 2020. Data were collected from the SWEDEHEART registry. Three other mandatory registers provided data on comorbidities, social factors and outcome variables. Adjusted Cox regression models were used to estimate mortality and morbidity. The median follow-up was 5 (0–11) years.

**Results:**

Current smokers were younger and had a higher proportion of previous myocardial infarction, heart failure, chronic respiratory disease, depression and low education compared with never smokers. Compared with never smokers, current smokers had higher risk for a major adverse cardiovascular event (MACE) (adjusted hazard ratio (aHR) 1.52, 95 % confidence interval (CI) 1.39–1.66), all-cause mortality (aHR 1.91, (1.71–2.14)) and stroke (aHR 1.49, (1.27–1.74)) but not for myocardial infarction (aHR 1.07, (0.91–1.26)). Compared with former smokers, current smokers had an increased risk for MACE (aHR 1.38, (1.27–1.49)), all-cause mortality (aHR 1.53, (1.39–1.69)), stroke (aHR 1.36, (1.18–1.56)), but not for myocardial infarction (aHR 1.15, (1.00–1.34)).

**Conclusions:**

There was a strong association between smoking and long-term risk for mortality and morbidity after CABG. The highest risks were observed in current smokers. The results emphasize the importance of motivating CABG patients to smoking cessation before considering CABG as a treatment option.

## Highlights

• Smoking at the time of CABG is associated with a markedly increased long-term risk for mortality and morbidity.
• Ex-smokers have longer survival after CABG than patients who smoke at the time of surgery.
• There are no sex-specific differences in long-term mortality after CABG related to smoking status at the time of surgery.

## 1 Introduction

Coronary artery disease (CAD) is the most common cause of death in both developing and developed countries [1]. In patients with complex multi-vessel and/or left main CAD, coronary artery bypass grafting (CABG) prolongs life and reduces angina symptoms [2].

There are more than one billion smokers worldwide [3]. Smoking is clearly associated with an increased risk of developing CAD [1]. However, the impact of smoking on survival after clinical manifestations of CAD, such as myocardial infarction, is debated. A smoker's paradox has been reported to exist, with unexpected favorable outcome after myocardial infarction and percutaneous coronary intervention (PCI) in smokers compared with never smokers [4,5]. On the other hand, patients who are smokers at the time of surgery are reported to have a higher incidence of early postoperative complications such as pulmonary complications and poor wound healing, as well as an increased risk of early mortality [[6], [7], [8]]. However, some studies have been unable to demonstrate a link between smoking and mortality [8,9], which leads to the question whether the smoker's paradox also exists in patients undergoing CABG.

Most previous studies in the field have focused on associations between smoking and early outcome after CABG [6,7]. Potential associations between smoking and long-term outcomes after CABG are less well investigated and the few studies that exist show contradictory results [6]. Furthermore, studies on sex-specific associations between smoking and outcome after CABG are lacking.

Considering the inconsistent results in long-term studies and the lack of knowledge regarding potential sex-specific differences, we designed a nationwide population-based cohort study aiming to explore the association between smoking and risk for long-term mortality, myocardial infarction, and stroke in men and women undergoing CABG.

## 2 Methods

### 2.1 Study population and data sources

Using the Swedish personal identity number, we merged data from four registries [10]. The Swedish Cardiac Surgery Registry [11], which is a part of the Swedish Web-system for Enhancement and Development of Evidence-Based Care in Heart Disease Evaluated According to Recommended Therapies (SWEDEHEART) registry, was used to identify 28,480 patients aged >18 years who underwent primary isolated CABG in Sweden in 2010–2020. Owing to missing data for smoking, 1,046 patients (3.7 %) were excluded, and the final study population consisted of 27,434 patients.

The Swedish Cardiac Surgery Registry was established in 1992 [11,12] and contains detailed data on patient characteristics, intraoperative variables and early complications for all open cardiac surgery procedures performed in Sweden since 1992. For the present study, the following preoperative variables were obtained from the registry: body mass index (BMI), left ventricular function (LVEF) (>50 %, 30–50 %, 20–30 %, <20 %) and smoking status. For the purposes of this study, the study population was divided into: (a) never smokers; (b) former smokers (>1 month); and (c) current smokers.

Information about other comorbidities was obtained from the National Patient Registry (NPR). Principal and secondary diagnoses for all hospitalizations in Sweden have been registered in the NPR since 1987, with 85–95 % validity [13,14]. The International Classification of Diseases, Ninth Revision (ICD-9), was used to define baseline comorbidities during the period 1987–1997 and the ICD-10 was used for data collected during 1997–2020. Comorbidities were collected from 1987 until the day of hospital admission*.* The ICD codes used are listed in Supplementary Table 1.

Data for socioeconomic variables were collected from the Longitudinal Integration Database for Health Insurance and Labor Market Studies (LISA) register held by Statistics Sweden [15]. The registry has annually collected socioeconomic information on all citizens aged >16 years in Sweden since 1990. Marital status at the year of surgery*,* was divided into four levels: (1) never been married; (2) married/cohabiting; (3) divorced; and (4) widowed. Length of education at the year of surgery*,* was stratified into three levels: (1) <10 years (compulsory school only); (2) 10–12 years (upper school); and (3) >12 years (college/university level). Income was measured as annual household disposable income at year of surgery, stratified into quintiles from Q1 (lowest income) to Q5 (highest income). The consumer price index according to Statistics Sweden (SCB) was used to adjust for inflation over time. If data were missing for the year of surgery, the latest information about education, marital status, and income was imputed from records for the most recent years before the surgery*.* The Cause of Death Register (CDR) provides information on causes of death in Sweden. The register has been updated annually since 1997 [16].

### 2.2 Outcome measures

We analyzed time to the first major adverse cardiovascular event (MACE) (defined as all-cause mortality, myocardial infarction, and stroke) and all-cause mortality, stroke and myocardial infarction separately. We also performed subgroup analyses in current smokers vs never smokers for MACE. In addition, we performed a sex-specific analysis for MACE and all-cause mortality, stroke and myocardial infarction. Follow-up for mortality and stroke started on the day of surgery, and for myocardial infarction at discharge.

### 2.3 Statistical methods

Descriptive, continuous variables are given as mean, standard deviation (SD), or median, minimum and maximum, and interquartile range (IQR), as appropriate. Categorical variables are described by frequency and percentage. For tests between three groups, the continuous variables (age, BMI and follow-up time) were tested by using Jonckheere-Terpstra test, not ordered categorical variables (civil status) were tested by using Chi-square test and binary and ordered categorical variables (all other variables, such as comorbidities) were tested by using Mantel-Haenszel Chi-square test. Crude event rates for the smoking status categories were calculated as number of events divided by the number of follow-up years per respective study group, and expressed as 100 person-years, with 95 % confidence intervals (CIs) estimated using exact Poisson limits. Cox regression was used to estimate the excess risk for outcomes in current vs former smokers, as well as for current and former smokers vs never smokers. Two models were estimated: the first adjusted for age and sex, and the second adjusted for age, sex, year of surgery, myocardial infarction, diabetes, hypertension, heart failure, atrial fibrillation, history of malignancy, hyperlipidaemia, previous stroke, chronic respiratory disease, peripheral vascular disease, renal insufficiency, depression, left ventricular function, BMI, marital status, education level and income. Subgroup analyses between never smokers and smokers were also conducted. The subgroups were defined by age (≤75 and > 75 years), sex, and a history or not of myocardial infarction, diabetes, hypertension, heart failure, atrial fibrillation, previous stroke, chronic respiratory disease, peripheral vascular disease, renal insufficiency and history of malignancy. Cumulative incidence functions were presented for MACE, stroke, MI, and death. For stroke and MI, the functions are adjusted for the competing risk of death by Aalen–Johansen estimators. For MACE and death, cumulative incidence was estimated as 1 minus the Kaplan–Meier estimator. A p-value of <0.05 was considered significant for the interaction term. All tests were two-tailed and analyses were performed in SAS software, version 9.4 (SAS Institute Inc, Cary, NC, USA).

### 2.4 Ethical considerations

The study was performed in line with the Declaration of Helsinki and approved by the Swedish Ethical Review Authority (registration number: 2021-00122). To ensure anonymity all personal identifiers were replaced by codes before analysis. The need for individual patient consent was waived by the review authority. The present paper follows the Strengthening the Reporting of Observational Studies in Epidemiology (STROBE) recommendations [17].

## 3 Results

### 3.1 Baseline characteristics

In total 27,434 patients who underwent isolated CABG were included (18.2 % women), mean age 67.9 years. Median follow-up time was 5 (range 0–11) years. Of the 27,434 patients, 8,593 (31.3 %) were never smokers, 14,666 (53.5 %) former smokers and 4,175 (15.2 %) current smokers. Baseline characteristics are presented in Table 1. Current smokers were younger and had a higher proportion of myocardial infarction, heart failure, chronic respiratory disease, peripheral vascular disease, and history of depression compared with former smokers and never smokers. Baseline characteristics for men and women are shown in Supplementary Tables 2 and 3 Altogether, 29.9 % of the men were never smokers, 55.4 % were former smokers and 14.7 % were current smokers. Among women, 37.5 % were never smokers, 44.7 % were former smokers and 17.8 % were current smokers. During follow-up, 4,260/27,434 (15.5 %) patients died, 1,209/8,593 (14.1 %) never smokers, 2,226/14,666 (15.2 %) former smokers and 825/4,175 (19.8 %) current smokers.

**Table 1:** Baseline characteristics in coronary artery bypass grafting (CABG) patients, by smoking status. Unless otherwise indicated, data are given as number and percentage.

	Total 27,434 n (%)	Never smokers 8,593 n (%)	Former smokers 14,666 n (%)	Current smokers 4,175 n (%)	P-value
Sex					<0.0001
Male	22,450 (81.8)	6,723 (78.2)	12,437 (84.8)	3,290 (78.8)	
Female	4,984 (18.2)	1,870 (21.8)	2,229 (15.2)	885 (21.2)	
Age, years, mean (SD)	67.9 (9.0)	68.6 (9.5)	68.8 (8.3)	63.4 (9.0)	<.0001
**Indication for surgery**					
Stable coronary artery disease	13,345 (48.6)	4,147 (48.3)	7,710 (52.6)	1,488 (35.6)	<0.0001
Unstable angina	7,706 (28.1)	2,264 (26.3)	4,379 (29.9)	1,063 (25.5)	0.42
Non-STEMI	9,641 (35.1)	2,729 (31.8)	5,152 (35.1)	1,760 (42.2)	<0.0001
STEMI	3,498 (12.8)	887 (10.3)	1,938 (13.2)	673 (16.1)	<0.0001
**Comorbidities**					
Myocardial infarction	13,568 (49.5)	3,829 (44.6)	7,228 (49.3)	2,511 (60.1)	<0.0001
Diabetes	8,317 (30.3)	2,135 (24.8)	4,905 (33.4)	1,277 (30.6)	<0.0001
Hypertension	17,981 (65.5)	5,379 (62.6)	10,189 (69.5)	2,413 (57.8)	0.18
Heart failure	3,512 (12.8)	874 (10.2)	1,984 (13.5)	654 (15.7)	<0.0001
Atrial fibrillation	2,644 (9.6)	807 (9.4)	1,558 (10.6)	279 (6.7)	0.0016
Previous stroke	1,862 (6.8)	488 (5.7)	1,109 (7.6)	265 (6.3)	0.0054
Chronic respiratory disease	2,551 (9.3)	500 (5.8)	1,481 (10.1)	570 (13.7)	<0.0001
Peripheral vascular disease	2,436 (8.9)	397 (4.6)	1,539 (10.5)	500 (12.0)	<0.0001
Renal insufficiency	1,177 (4.3)	329 (3.8)	714 (4.9)	134 (3.2)	0.81
History of cancer	4,288 (15.6)	1,411 (16.4)	2,428 (16.6)	449 (10.8)	<0.0001
Hyperlipidaemia	10,182 (37.1)	2,873 (33.4)	5,854 (39.9)	1,455 (34.9)	<0.0001
History of depression	1,534 (5.6)	325 (3.8)	774 (5.3)	435 (10.4)	<0.0001
**Left ventricular function**					<0.0001
Normal (>50 %)	17,440 (69.1)	5,754 (74.1)	9,435 (69.1)	2,251 (59.3)	
31–50 %	6,416 (25.4)	1,711 (22.0)	3,512 (25.7)	1,193 (31.4)	
21–30 %	1,159 (4.6)	259 (3.3)	610 (4.5)	290 (7.6)	
<20 %	206 (0.8)	39 (0.5)	105 (0.8)	62 (1.6)	
**BMI, kg/m^2^, mean (SD)**	27.8 (6.8)	27.3 (7.3)	28.1 (7.1)	27.5 (4.7)	<0.0001
**Marital status**					<0.0001
Never been married	3,866 (14.1)	1,228 (14.3)	1,791 (12.2)	847 (20.3)	
Married/cohabiting	16,405 (59.8)	5,366 (62.4)	9,092 (62.0)	1,947 (46.6)	
Divorced	5,049 (18.4)	1,230 (14.3)	2,675 (18.2)	1,144 (27.4)	
Widowed	2,110 (7.7)	796 (8.9)	1,105 (7.5)	236 (5.7)	
**Education**					<0.0001
<10 years	8,973 (33.1)	2,503 (29.4)	4,874 (33.6)	1,596 (38.9)	
10–12 years	11,922 (44.0)	3,540 (41.6)	6,497 (44.8)	1,885 (45.9)	
>12 years	6,218 (22.9)	2,465 (29.0)	3,131 (21.6)	622 (15.2)	
**Income**					<0.0001
Q1 (Lowest income)	5,495 (20.0)	1,563 (18.2)	2,640 (18.0)	1,292 (30.9)	
Q2	5,481 (20.0)	1,665 (19.4)	2,937 (20.0)	879 (21.1)	
Q3	5,486 (20.0)	1,650 (19.2)	3,127 (21.3)	709 (17.0)	
Q4	5,487 (20.0)	1,739 (20.2)	3,037 (20.7)	711 (17.0)	
Q5 (Highest income)	5,485 (20.0)	1,976 (23.0)	2,925 (19.9)	584 (14.0)	

BMI = body mass index; Q1–Q5 = quintiles 1–5; SD = standard deviation; STEMI = ST-segment elevation myocardial infarction.

### 3.2 Cumulative incidence and survival probability by smoking status

**Fig. 1:**
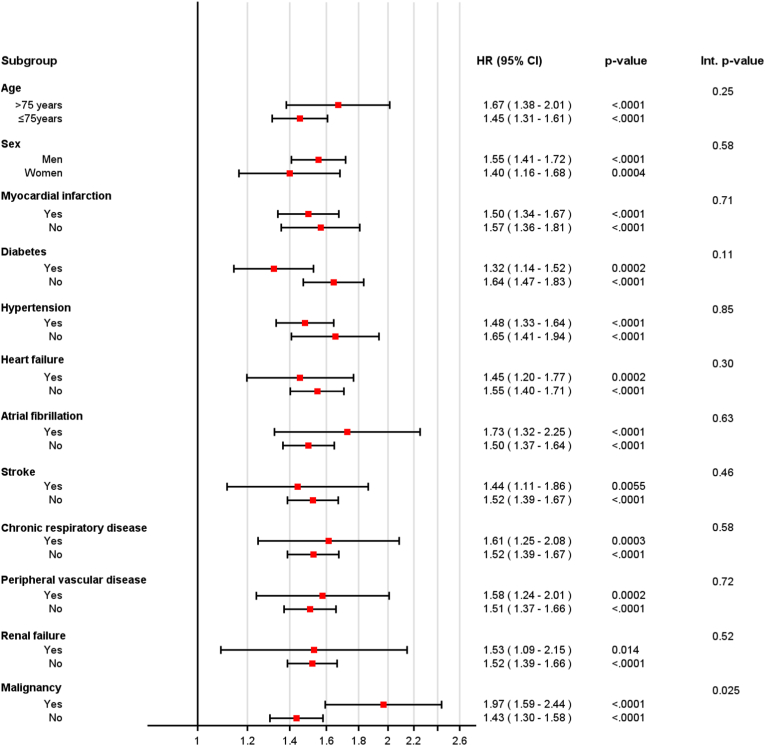
Cumulative *incidence* for major adverse cardiovascular event (MACE), all-cause mortality, stroke and myocardial infarction, by smoking status at the time of coronary artery bypass grafting (CABG). The incidence curves for stroke and myocardial infarction are adjusted for death as competing risk*.*

Cumulative incidence for MACE, stroke and myocardial infarction is shown in Fig. 1. The overall cumulative incidence of MACE at 10 years was 37 % (95 % CI 35–39 %) in never smokers, 42 % (95 % CI 40–43 %) in former smokers and 45 % (95 % CI 42–47 %) in current smokers (p < 0.001). The cumulative incidence at 10 years for all-cause mortality was 27 % (95 % CI 25–28 %) in never smokers, 31 % (95 % CI 29–32 %) in former smokers and 33 % (95 % CI 31–36 %) in current smokers.

### 3.3 Risk for mortality and morbidity by smoking status

Overall, the incidence rates for MACE, all-cause mortality, stroke and myocardial infarction were higher in former smokers and current smokers than in never smokers (Table 2). Compared with never smokers, former smokers had a higher risk for MACE (adjusted hazard ratio (aHR) 1.10, 95 % CI -1.03–1.18) and all-cause mortality (aHR 1.25, 95 % CI 1.15–1.36). The risks for stroke (aHR 1.10, 95 % CI 0.98–1.23) and myocardial infarction (aHR 0.93, 95 % CI 0.82–1.05) did not differ significantly. Compared with never smokers, current smokers had a higher risk for MACE (aHR 1.52, 95 % CI 1.39–1.66), all-cause mortality (aHR 1.91, 95 % CI 1.71–2.14) and stroke (aHR 1.49, 95 % CI 1.27–1.74) but not for myocardial infarction (aHR 1.07, 95 % CI 0.91–1.26).

**Table 2:** Incidence rate, and unadjusted and multi-adjusted hazard ratio by smoking status in coronary artery bypass grafting (CABG) patients.

	No. of patients	No. of events	IR per 100 person-years (95 % CI)	Unadjusted hazard ratio∗ (95 % CI)	Adjusted hazard ratio∗∗ (95 % CI)
**MACE**					
Never smokers	8,593	1,943	4.40 (4.21–4.60)	(ref)	(ref)
Former smokers	14,666	3,462	5.20 (5.03–5.37)	1.24 (1.17–1.31)	1.10 (1.03–1.18)
Current smokers	4,175	1,245	6.32 (5.97–6.68)	1.98 (1.684–2.13)	1.52 (1.39–1.66)
**All-cause mortality**					
Never smokers	8,593	1,209	2.52 (2.38–2.66)	(ref)	(ref)
Former smokers	14,666	2,226	3.06 (2.93–3.19)	1.38 (1.29–1.48)	1.25 (1.15–1.36)
Current smokers	4,175	825	3.75 (3.50–4.01)	2.54 (2.31–2.78)	1.91 (1.71–2.14)
**Stroke**					
Never smokers	8,593	638	1.39 (1.28–1.50)	(ref)	(ref)
Former smokers	14,666	1,131	1.63 (1.54–1.73)	1.20 (1.09–1.32)	1.10 (0.98–1.23)
Current smokers	4,175	371	1.79 (1.61–1.98)	1.78 (1.56–2.03)	1.49 (1.27–1.74)
**Myocardial infarction**					
Never smokers	8,593	566	1.23 (1.13–1.34)	(ref)	(ref)
Former smokers	14,666	926	1.33 (1.24–1.41)	1.10 (0.99–1.22)	0.93 (0.82–1.05)
Current smokers	4,175	340	1.63 (1.47–1.82)	1.39 (1.21–1.59)	1.07 (0.91–1.26)

∗Adjusted for sex and age. ∗∗Adjusted for sex, age, year of surgery, myocardial infarction, diabetes, hypertension, heart failure, atrial fibrillation, history of cancer, hyperlipidaemia, previous stroke, chronic respiratory disease, peripheral vascular disease, renal insufficiency, depression, left ventricular function, body mass index (BMI), marital status, education level, income level.

CI = confidence interval; IR = incidence rate, MACE = major adverse cardiovascular event.

Table 3 shows the adjusted hazard ratio for former compared with current smokers. Compared with former smokers, current smokers had a higher risk for MACE (aHR 1.38, 95 % CI 1.27–1.49), all-cause mortality (aHR 1.53, 95 % CI 1.39–1.69), stroke (aHR 1.36, 95 % CI 1.18–1.56), but not significantly for myocardial infarction (aHR 1.15, 95 % CI 1.00–1.34, p = 0.06).

**Table 3:** Unadjusted and multi-adjusted hazard ratio in former smokers compared with current smokers.

	No. of patients	No. of events	Unadjusted hazard ratio∗ (95 % CI)	Adjusted hazard ratio∗∗ (95 % CI)
**MACE**				
Former smokers	14,666	3,462	ref	ref
Current smokers	4,175	1,245	1.59 (1.49–1.70)	1.38 (1.27–1.49)
**All-cause mortality**				
Former smokers	14,666	2,226	ref	ref
Current smokers	4,175	825	1.84 (1.69–2.00)	1.53 (1.39–1.69)
**Stroke**				
Former smokers	14,666	1,131	ref	ref
Current smokers	4,175	371	1.48 (1.31–1.67)	1.36 (1.18–1.56)
**Myocardial infarction**				
Former smokers	14,666	926	ref	ref
Current smokers	4,175	340	1.26 (1.11–1.43)	1.15 (1.00–1.34)

∗Adjusted for sex and age. ∗∗Adjusted for sex, age, year of surgery, myocardial infarction, diabetes, hypertension, heart failure, atrial fibrillation, history of cancer, hyperlipidaemia, previous stroke, chronic respiratory disease, peripheral vascular disease, renal insufficiency, depression, left ventricular function, body mass index (BMI), marital status, education level, income level. CI = confidence interval; IR = incidence rate; MACE = major adverse cardiovascular event.

### 3.4 Subgroup analyses in current smokers vs never smokers

**Fig. 2:**
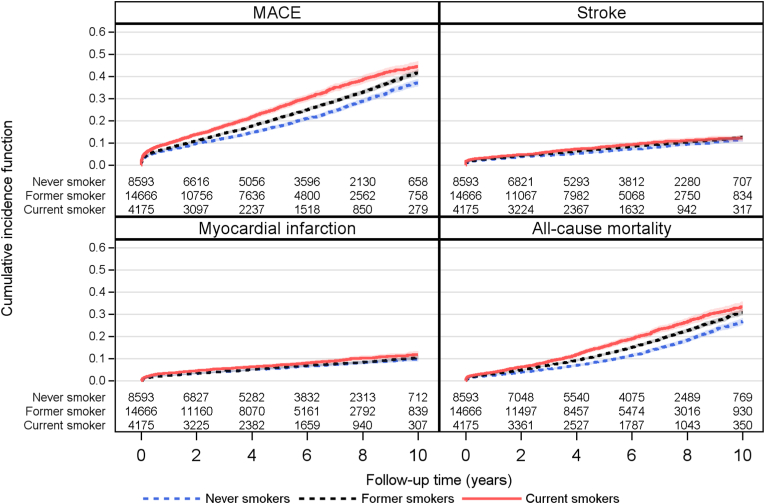
Hazard ratio and interactions with risk for a major adverse cardiovascular event (MACE) in current smokers vs never smokers. CI = confidence interval; HR = hazard ratio; Int. p-value = interactions p-value.

Fig. 2 shows subgroup analyses for MACE in smokers vs never smokers. Current smoking was associated with increased MACE risk in all investigated subgroups. For history of cancer, the association was even stronger and a significant interaction was seen between current smoking and MACE in patients with history of malignancy (p = 0.025).

### 3.5 Risks in men and women by smoking status at time of surgery

In separate analyses the risks for MACE, all-cause mortality, stroke and myocardial infarction in men and women were explored (Supplementary Table 4). In men, current smoking, compared with never smoking, was associated with an increased risk for MACE (aHR 1.55, 95 % CI 1.41–1.72), all-cause mortality (aHR 1.96, 95 % CI 1.72–2.23) and stroke (aHR 1.48, 95 % CI 1.24–1.77) but not for myocardial infarction (aHR 1.18, 95 % CI 0.98–1.43). In women, current smoking, compared with never smoking, was associated with an increased risk for MACE (aHR 1.40, 95 % CI 1.16–1.68), all-cause mortality (aHR 1.73, 95 % CI 1.38–2.17) and stroke (aHR 1.36, 95 % CI 1.10–2.16), but not for myocardial infarction (aHR 0.85, 95 % CI 0.61–1.19). There were no significant interactions between men and women for MACE, all-cause mortality, stroke or myocardial infarction.

## 4 Discussion

In this large nationwide population-based observational study, the main findings were that: (1) smoking at the time of surgery and former smoking were strongly associated with worse long-term outcome after CABG, compared with never smoking. (2) The highest risk was observed in patients who were smoking at the time of surgery. (3) There were no sex differences in the risks of MACE, all-cause mortality, stroke or myocardial infarction in current smokers*.* (4) The risks were also consistent in subgroups based on age and comorbidities, except for history of malignancy in current smokers.

In the present study, current smoking and former smoking were clearly associated with increased long-term mortality after CABG. Our results are in line with other studies. Saxena et al. reported increased long-term risk for mortality among active smokers compared with never smokers [8] and Papathanasiou et al. showed that patients who were smoking at the time of surgery and who continued smoking after surgery had about twofold increased risk of mortality compared with never smokers [2]. Abreu et al. recently reported that ex-smokers had similar long-term survival as never smokers, while patients smoking at the time of CABG had increased long-term mortality risk [18]. However, there are studies showing conflicting results. For example, Tang et al. found no association between current smoking and in-hospital mortality [19] and a recent study by Shahin et al. found no difference in long-term mortality between current smokers and never smokers [20].

It has been suggested that a smoker's paradox exists. The term refers to a favorable outcome for smokers with cardiovascular diseases and was first introduced about 30 years ago after studies showed that smokers with acute myocardial infarction experienced lower mortality compared with never smokers [21,22]. Gupta et al. found, in a large registry study including 985,174 patients with ST-segment elevation myocardial infarction undergoing primary PCI, that smokers had lower in-hospital mortality compared with never smokers (adjusted odds ratio 0.60, 95 % CI 0.58–0.62) [22]. By contrast, in a recent large, patient-level, pooled meta-analysis with 5-year follow-up, smoking was demonstrated to be an important predictor of adverse outcomes after PCI [21]. The results of the present study argue strongly against the existence of a smoker's paradox in patients undergoing CABG.

Few previous studies have explored the long-term risk for MACE, stroke and myocardial infarction after CABG. When current smokers were compared to never smokers, there were significant differences in the adjusted risk for MACE, all-cause death and stroke, but not for myocardial infarction. This should however not be interpreted that smoking is not harmful to the coronary arteries. Myocardial infarction was a secondary endpoint, the unadjusted risk was higher for smokers and the incidence of myocardial infarction during follow-up was so low (approx. 1.3 % per year) that there is a clear risk for a statistical type II error. Interestingly*,* Saxena et al. observed that current smokers showed a lower risk for early postoperative myocardial infarction after *CABG* compared with never smokers [8]. However, one study published almost three decades ago showed that patients who continued smoking after CABG had an increased risk for myocardial infarction [23]. Also, Zhang et al. found that smoking, when used as a time-dependent variable, was associated with myocardial infarction. This revealed that patients who continued to smoke or relapsed to smoking after surgery had an increased risk for myocardial infarction [24]. These results support the need to explore changes in smoking patterns after surgery, and to encourage cessation. In the present study, a stronger association was observed between smoking and MACE in patients with, compared to without, a history of malignancy. The finding should, however, be interpreted with caution given the limited number of smoking patients with a history of malignancy. Nevertheless, it is of outmost importance that both patients with and without a history of malignancy avoid smoking after CABG*.*

The results from the present study show that, compared with never smokers, the risk for stroke was increased in current smokers but not in former smokers. This is a novel and interesting finding, as a previous study by Saxena et al. reported that smoking was not associated with increased risk of stroke [8]. However, a recent case-control study in a stroke population reported that smoking was associated with an increased risk of acute stroke [25].

Smoking cessation after CABG is strongly associated with improved survival [2,26,27]. However, a study by Ampatzidou et al. showed that patients who underwent CABG initially stopped smoking but that >50 % of those who were current smokers at the time of surgery could not maintain the smoking cessation [28]. There is a lack of knowledge about how patients undergoing CABG manage to quit smoking and remain smoke-free before and after surgery. Previous studies regarding smoking cessation and CABG reported that <50 % of patients who were current smokers at the time of surgery wanted to participate in studies regarding their smoking habits [27,28]. Furthermore, Paryad et al. investigated patients' adherence to smoking cessation based on their illness perception after CABG. The study showed that despite the patients' understanding of their health status they were not convinced to stop smoking [27]. Ampatzidou et al. reported that although their study's patients were aware of the risk of any postoperative complications linked to smoking, this still did not strengthen their motivation for smoking cessation [28]. This, together with the results of the present study, shows that patients who are smokers at the time of surgery need better support for smoking cessation and relapse prevention. According to guideline recommendations regarding patients with acute coronary syndrome, the support for sustainable smoking cessation should begin during hospitalization. The support should include a combination of counselling, drug interventions such as nicotine replacement therapy, and behavioral support [29]. This recommendation should be more marked in future guidelines and in the clinical management of patients undergoing CABG.

To our knowledge, no previous study has explored sex-specific associations between smoking and risk for long-term mortality and morbidity following CABG. In the present study we could not observe any sex-specific differences in outcome related to smoking status between men and women. This important finding supports previous research on sex-specific differences in mortality after CABG in general, which, though reporting a worse unadjusted prognosis for women shows that, after adjustment for confounders such as comorbidities and social factors, the risk is no longer significant [[30], [31], [32], [33]].

This study has both strengths and limitations. The strengths include the large nationwide study population, the real-life data and the complete follow-up on survival for 10 years. The databases that were used to collect the data are mandatory and validated. Few studies have previously investigated the long-term risk for mortality and morbidity after CABG. The large sample size allowed the analysis of men and women separately.

Limitations include the retrospective design, which implies an underlying risk of residual confounding and selection bias. Because of limitations in the registers, we started the follow-up for myocardial infarction at discharge from hospital, which may have resulted in an underestimation of events. We could not *adjust* for *use of medication or* lifestyle variables such as diet, physical activity, or stress*,* in our statistical models. The group of former smokers consisted of patients who stopped smoking from several years before surgery, to patients who stopped 1 month before surgery. The time since smoking cessation may affect the patients’ cardiovascular risk*.* Furthermore, we do not know whether patients started to smoke again during the follow-up period.

## 5 Conclusion

In conclusion, there is a strong association between smoking status at the time of CABG and long-term risks for mortality and morbidity after CABG. The highest risk was observed in patients smoking at the time of surgery. The results emphasize the importance of encouraging and motivating patients with CAD to stop smoking even before CABG becomes an option. Further research is needed to investigate patients’ reported data and reasons for continuing smoking and which factors are important and provide motivation for successful and sustainable smoking cessation.

## CRediT authorship contribution statement

**Emelie Johansson:** Writing – review & editing, Writing – original draft, Project administration, Investigation, Formal analysis, Data curation, Conceptualization. **Malin Stenman:** Writing – review & editing, Writing – original draft, Supervision, Methodology. **Emma C. Hansson:** Writing – review & editing, Writing – original draft, Supervision, Methodology. **Carl-Johan Malm:** Writing – review & editing, Writing – original draft, Methodology. **Sossio Perrotta:** Writing – review & editing, Writing – original draft, Methodology. **Aldina Pivodic:** Writing – review & editing, Writing – original draft, Validation, Software, Methodology, Investigation, Formal analysis, Data curation. **Anders Jeppsson:** Writing – review & editing, Writing – original draft, Validation, Supervision, Methodology, Data curation, Conceptualization. **Susanne J. Nielsen:** Writing – review & editing, Writing – original draft, Validation, Supervision, Project administration, Methodology, Investigation, Funding acquisition, Formal analysis, Data curation, Conceptualization.

## Disclosures

AJ has received fees for consultancy and/or lectures from AstraZeneca, Werfen, Novo Nordisk, Bayer, Boehringer Ingelheim, Pharmacosmos, and LFB Biotechnologies outside the present work.

## Data availability

Data will be shared on reasonable request to the corresponding author if permissions are obtained from SWEDEHEART and the Swedish National Board of Health and Welfare.

## Declaration of competing interest

All authors are requested to disclose any actual or potential conflict of interest including any financial, personal or other relationships with other people or organizations within three years of beginning the submitted work that could inappropriately influence, or be perceived to influence, their work. If there are no conflicts of interest, the COI should read: “The authors report no relationships that could be construed as a conflict of interest”.
