# Supplementary material for: Smoking and long-term risks for morbidity and mortality after coronary artery bypass grafting
Source: Int J Cardiol Cardiovasc Risk Prev. 2025 Sep 8;27:200512. doi: 10.1016/j.ijcrp.2025.200512 (PMC12744804; doi:10.1016/j.ijcrp.2025.200512)
Supplement: Multimedia component 1 [file mmc1.docx]

**Supplementary Table 1.** International Classification of Diseases (ICD) codes for baseline characteristics.

|  | **ICD-9**  **codes** | **ICD-10**  **codes** |
| --- | --- | --- |
| Myocardial infarction | 410 | I21 |
| Diabetes | 250 | E10–E14 |
| Hypertension | 401–405 | I10–I15 |
| Heart failure | 428 | I50 |
| Atrial fibrillation | 427D | I48 |
| Previous stroke | 431–434, 436 | I61–I64, I69 |
| Chronic respiratory disease | 490–496 | J40–J47 |
| Peripheral vascular disease | 440–444, 447 | I65–I65.9, I70–174, I77 |
| Renal insufficiency | 584–586 | N17–N19 |
| Malignancy | 140–208 | C00–C97 |
| Depression | 296, 311, 300E | F3 |
| Hyperlipidemia | – | E78 |

**Supplementary Table 2.** Baseline characteristics in men undergoing coronary artery bypass grafting (CABG), by smoking status. Unless otherwise indicated, data are given as number and percentage.

|  | **Total**  **n=22,450**  **n (%)** | **Never smoker n=6,723**  **n (%)** | **Former smoker n=12,437**  **n (%)** | **Current smoker**  **n=3,290**  **n (%)** | **P-value** |
| --- | --- | --- | --- | --- | --- |
| Age, yrs, mean (SD) | 67.6 (9.0) | 67.8 (9.5) | 68.6 (8.3) | 63.0 (9.0) | <.0001 |
| **Indication for surgery** |  |  |  |  |  |
| Stable coronary artery disease | 11,034 (49.1) | 3,248 (48.3) | 6,585 (52.9) | 1,201 (36.5) | <.0001 |
| Unstable angina | 6,244 (27.8) | 1,731 (25.7) | 3,684 (29.6) | 829 (25.2) | 0.23 |
| Non-STEMI | 7,663 (34.1) | 2,039 (30.3) | 4,270 (34.3) | 1,354 (41.2) | <.0001 |
| STEMI | 2,921 (13.0) | 718 (10.7) | 1,670 (13.4) | 533 (16.2) | <.0001 |
| **Comorbidities** |  |  |  |  |  |
| Myocardial infarction | 10,928 (48.7) | 2,906 (43.2) | 6,074 (48.8) | 1,948 (59.2) | <.0001 |
| Diabetes | 6,586 (29.3) | 1,542 (22.9) | 4,072 (32.7) | 972 (29.5) | <.0001 |
| Hypertension | 14,361 (64.0) | 4,012 (59.7) | 8,511 (68.4) | 1,838 (55.9) | 0.64 |
| Heart failure | 2,831 (12.6) | 656 (9.8) | 1,660 (13.3) | 515 (15.7) | <.0001 |
| Atrial fibrillation | 2,210 (9.8) | 641 (9.5) | 1,342 (10.8) | 227 (6.9) | 0.0088 |
| Previous stroke | 1,517 (6.8) | 376 (5.6) | 931 (7.5) | 210 (6.4) | 0.0070 |
| Chronic respiratory disease | 1,860 (8.3) | 347 (5.2) | 1,108 (8.9) | 405 (12.3) | <.0001 |
| Peripheral vascular disease | 1,885 (8.4) | 284 (4.2) | 1,218 (9.8) | 383 (11.6) | <.0001 |
| Renal insufficiency | 938 (4.2) | 251 (3.7) | 587 (4.7) | 100 (3.0) | 0.70 |
| History of cancer | 3,510 (15.6) | 1,108 (16.5) | 2,061 (16.6) | 341 (10.4) | <.0001 |
| Hyperlipidaemia | 8,198 (36.5) | 2,193 (32.6) | 4,889 (39.3) | 1,116 (33.9) | 0.0002 |
| Depression | 1,120 (5.0) | 229 (3.4) | 588 (4.7) | 303 (9.2) | <.0001 |
| **Left ventricular function** |  |  |  |  | <.0001 |
| Normal (>50%) | 14,154 (68.5) | 4,472 (73.7) | 7,940 (68.5) | 1,742 (58.2) |  |
| 31–50% | 5,339 (25.9) | 1,362 (22.4) | 3,018 (26.1) | 959 (32.0) |  |
| 21–30% | 977 (4.7) | 209 (3.4) | 530 (4.6) | 238 (7.9) |  |
| <20% | 178 (0.9) | 27 (0.4) | 96 (0.8) | 55 (1.8) |  |
| **BMI**, kg/m^2^, mean (SD) | 27.7 (6.5) | 27.3 (7.8) | 28.1 (6.2) | 27.5 (4.6) | <.0001 |
| **Marital status** |  |  |  |  | <.0001 |
| Never been married | 3,321 (14.8) | 1,057 (15.7) | 1,552 (12.5) | 712 (21.6) |  |
| Married/cohabiting | 13,996 (62.4) | 4,405 (65.5) | 8,004 (64.4) | 1,587 (48.3) |  |
| Divorced | 4,007 (17.9) | 942 (14.0) | 2,191 (17.6) | 874 (26.6) |  |
| Widowed | 1,122 (5.0) | 319 (4.7) | 687 (5.5) | 116 (3.5) |  |
| **Education** |  |  |  |  | <.0001 |
| <10 years | 7,153 (32.2) | 1,835 (27.4) | 4,080 (33.2) | 1,238 (38.3) |  |
| 10–12 years | 9,784 (44.0) | 2,800 (41.8) | 5,511 (44.8) | 1,473 (45.5) |  |
| >12 years | 5,290 (23.8) | 2,057 (30.7) | 2,709 (22.0) | 524 (16.2) |  |
| **Income** |  |  |  |  | <.0001 |
| Q1 (Lowest income) | 3,813 (17.0) | 908 (13.5) | 1,929 (15.5) | 976 (29.7) |  |
| Q2 | 4,379 (19.5) | 1,245 (18.5) | 2,455 (19.7) | 679 (20.6) |  |
| Q3 | 4,569 (20.4) | 1,309 (19.5) | 2,684 (21.6) | 576 (17.5) |  |
| Q4 | 4,739 (21.1) | 1,487 (22.1) | 2,686 (21.6) | 566 (17.2) |  |
| Q5 (Highest income) | 4,950 (22.0) | 1,774 (26.4) | 2,683 (21.6) | 493 (15.0) |  |
| BMI = body mass index; Q1–Q5 = quintiles 1–5; SD = standard deviation; STEMI = ST-segment elevation myocardial infarction. | | | | |  |

**Supplementary Table 3.** Baseline characteristics in women undergoing coronary artery bypass grafting (CABG), by smoking status**.** Unless otherwise indicated, data are given as number and percentage.

| **Variable** | **Total**  **n=4,984**  **n (%)** | **Never smoker**  **n=1,870**  **n (%)** | **Former smoker**  **n=2,229**  **n (%)** | **Smoker**  **n=885**  **n (%)** | **P-value** |
| --- | --- | --- | --- | --- | --- |
| Age, yrs, mean (SD) | 69.4 (9.0) | 71.3 (9.3) | 69.7 (8.1) | 64.9 (8.9) | <.0001 |
| **Indication for surgery** |  |  |  |  |  |
| Stable coronary artery disease | 2,311 (46.4) | 899 (48.1) | 1,125 (50.5) | 287 (32.4) | <.0001 |
| Unstable angina | 1,462 (29.3) | 533 (28.5) | 695 (31.2) | 234 (26.4) | 0.66 |
| Non-STEMI | 1,978 (39.7) | 690 (36.9) | 882 (39.6) | 406 (45.9) | <.0001 |
| STEMI | 577 (11.6) | 169 (9.0) | 268 (12.0) | 140 (15.8) | <.0001 |
| **Comorbidities** |  |  |  |  |  |
| Myocardial infarction | 2,640 (53.0) | 923 (49.4) | 1,154 (51.8) | 563 (63.6) | <.0001 |
| Diabetes | 1,731 (34.7) | 593 (31.7) | 833 (37.4) | 305 (34.5) | 0.025 |
| Hypertension | 3,620 (72.6) | 1,367 (73.1) | 1,678 (75.3) | 575 (65.0) | 0.0007 |
| Heart failure | 681 (13.7) | 218 (11.7) | 324 (14.5) | 139 (15.7) | 0.0014 |
| Atrial fibrillation | 434 (8.7) | 166 (8.9) | 216 (9.7) | 52 (5.9) | 0.048 |
| Previous stroke | 345 (6.9) | 112 (6.0) | 178 (8.0) | 55 (6.2) | 0.38 |
| Chronic respiratory disease | 691 (13.9) | 153 (8.2) | 373 (16.7) | 165 (18.6) | <.0001 |
| Peripheral vascular disease | 551 (11.1) | 113 (6.0) | 321 (14.4) | 117 (13.2) | <.0001 |
| Renal insufficiency | 239 (4.8) | 78 (4.2) | 127 (5.7) | 34 (3.8) | 0.76 |
| History of cancer | 778 (15.6) | 303 (16.2) | 367 (16.5) | 108 (12.2) | 0.025 |
| Hyperlipidaemia | 1,984 (39.8) | 680 (36.4) | 965 (43.3) | 339 (38.3) | 0.039 |
| Depression | 414 (8.3) | 96 (5.1) | 186 (8.3) | 132 (14.9) | <.0001 |
| **Left ventricular function** |  |  |  |  | <.0001 |
| Normal (>50%) | 3,286 (71.9) | 1,282 (75.7) | 1,495 (71.9) | 509 (63.5) |  |
| 31–50% | 1,077 (23.6) | 349 (20.6) | 494 (23.8) | 234 (29.2) |  |
| 21–30% | 182 (4.0) | 50 (3.0) | 80 (3.8) | 52 (6.5) |  |
| >20% | 28 (0.6) | 12 (0.7) | 9 (0.4) | 7 (0.9) |  |
| **BMI,** kg/m^2^, mean (SD) | 27.9 (8.1) | 27.3 (4.9) | 28.5 (10.7) | 27.3 (4.9) | 0.025 |
| **Marital status** |  |  |  |  | <.0001 |
| Never been married | 545 (10.9) | 171 (9.1) | 239 (10.7) | 135 (15.3) |  |
| Married/cohabiting | 2,409 (48.3) | 961 (51.4) | 1,088 (48.8) | 360 (40.7) |  |
| Divorced | 1,042 (20.9) | 288 (15.4) | 484 (21.7) | 270 (30.5) |  |
| Widowed | 988 (19.8) | 450 (24.1) | 418 (18.8) | 120 (13.6) |  |
| **Education** |  |  |  |  | <.0001 |
| <10 years | 1,820 (37.2) | 668 (36.8) | 794 (36.1) | 358 (41.2) |  |
| 10–12 years | 2,138 (43.8) | 740 (40.7) | 986 (44.8) | 412 (47.5) |  |
| <12 years | 928 (19.0) | 408 (22.5) | 422 (19.2) | 98 (11.3) |  |
| **Income** |  |  |  |  | 0.51 |
| Q1 (Lowest income) | 1,682 (33.7) | 655 (35.0) | 711 (31.9) | 316 (35.7) |  |
| Q2 | 1,102 (22.1) | 420 (22.5) | 482 (21.6) | 200 (22.6) |  |
| Q3 | 917 (18.4) | 341 (18.2) | 443 (19.9) | 133 (15.0) |  |
| Q4 | 748 (15.0) | 252 (13.5) | 351 (15.7) | 145 (16.4) |  |
| Q5 (Highest income) | 535 (10.7) | 202 (10.8) | 242 (10.9) | 91 (10.3) |  |
| BMI = body mass index; Q1–Q5 = quintiles 1–5; SD = standard deviation; STEMI = ST-segment elevation myocardial infarction. | | | | |  |

**Supplementary Table 4.** Number of events and hazard ratio in men and women who had undergone coronary artery bypass grafting (CABG), by smoking status.

|  | **Men** | | | **Women** | | |  |
| --- | --- | --- | --- | --- | --- | --- | --- |
|  | **No. of patients/**  **No. of events** | **Unadjusted hazard ratio***  **(95% CI)** | **Adjusted hazard ratio****  **(95% CI)** | **No. of patients/ No. of events** | **Unadjusted hazard ratio* (95% CI)** | **Adjusted hazard ratio****  **(95% CI)** | **Interaction with sex**  **p-value** |
| **MACE** |  |  |  |  |  |  |  |
| Never smokers | 6,723/1,403 | (ref) | (ref) | 1,870/540 | (ref) | (ref) | 0.58 |
| Former smokers | 12,437/2,854 | 1.25 (1.17–1.33) | 1.10 (1.03–1.19) | 2,229/608 | 1.19 (1.06–1.34) | 1.10 (0.96–1.27) |  |
| Current smokers | 3,290/957 | 2.05 (1.88–2.23) | 1.55 (1.41–1.72) | 885/288 | 1.74 (1.49–2.02) | 1.40 (1.16–1.68) |  |
| **All-cause mortality** |  |  |  |  |  |  |  |
| Never smokers | 6,723/852 | (ref) | (ref) | 1,870/357 | (ref) | (ref) | 0.97 |
| Former smokers | 12,437/1837 | 1.39 (1.28–1.51) | 1.26 (1.15–1.39) | 2,229/389 | 1.29 (1.12–1.50) | 1.18 (1.00–1.41) |  |
| Current smokers | 3,290/625 | 2.62 (2.35–2.91) | 1.96 (1.72–2.23) | 885/200 | 2.22 (1.85–2.67) | 1.73 (1.38–2.17) |  |
| **Stroke** |  |  |  |  |  |  |  |
| Never smokers | 6,723/466 | (ref) | (ref) | 1,870/172 | (ref) | (ref) | 0.83 |
| Former smokers | 12,437/944 | 1.21 (1.08–1.35) | 1.09 (0.96–1.24) | 2,229/187 | 1.13 (0.91–1.39) | 1.13 (0.88–1.45) |  |
| Current smokers | 3,290/285 | 1.82 (1.56–2.11) | 1.48 (1.24–1.77) | 885/86 | 1.60 (1.21–2.10) | 1.36 (1.10–2.16) |  |
| **Myocardial infarction** |  |  |  |  |  |  |  |
| Never smokers | 6,723/378 | (ref) | (ref) | 1,870/188 | (ref) | (ref) | 0.052 |
| Former smokers | 12,437/720 | 1.14 (1.00–1.29) | 0.93 (0.81–1.08) | 2,229/206 | 1.05 (0.86–1.28) | 0.97 (0.76–1.23) |  |
| Current smokers | 3,290/258 | 1.53 (1.30–1.79) | 1.18 (0.98–1.43) | 885/82 | 1.10 (0.84–1.44) | 0.85 (0.61–1.19) |  |

*Adjusted for sex and age. **Adjusted for sex, age, year of surgery, myocardial infarction, diabetes, hypertension, heart failure, atrial fibrillation, history of cancer, hyperlipidaemia, previous stroke, chronic respiratory disease, peripheral vascular disease, renal insufficiency, depression, left ventricular function, body mass index (BMI), marital status, education level, income level. CI = confidence interval; MACE = major adverse cardiovascular event.
